# Detection of Corona Faults in Switchgear by Using 1D-CNN, LSTM, and 1D-CNN-LSTM Methods

**DOI:** 10.3390/s23063108

**Published:** 2023-03-14

**Authors:** Yaseen Ahmed Mohammed Alsumaidaee, Chong Tak Yaw, Siaw Paw Koh, Sieh Kiong Tiong, Chai Phing Chen, Talal Yusaf, Ahmed N Abdalla, Kharudin Ali, Avinash Ashwin Raj

**Affiliations:** 1 College of Graduate Studies (COGS), Universiti Tenaga Nasional (The Energy University), Jalan Ikram-Uniten, Kajang 43000, Selangor, Malaysia; 2 Institute of Sustainable Energy, Universiti Tenaga Nasional (The Energy University), Jalan Ikram-Uniten, Kajang 43000, Selangor, Malaysia; 3 Department Electrical and Electronics Engineering, Universiti Tenaga Nasional (The Energy University), Jalan Ikram-Uniten, Kajang 43000, Selangor, Malaysia; 4 School of Engineering and Technology, Central Queensland University, Brisbane, QLD 4009, Australia; 5 Faculty of Electronic Information Engineering, Huaiyin Institute of Technology, Huai’an 223003, China; 6 Faculty of Electrical and Automation Engineering Technology, UC TATI, Teluk Kalong, Kemaman 24000, Terengganu, Malaysia; 7 Tenaga National Berhard Research Sdn. Bhd., No. 1, Kawasan Institusi Penyelidikan, Jln Ayer Hitam, Kajang 43000, Selangor, Malaysia

**Keywords:** corona discharge, energy, switchgear, 1D-CNN-LSTM, faults

## Abstract

The damaging effects of corona faults have made them a major concern in metal-clad switchgear, requiring extreme caution during operation. Corona faults are also the primary cause of flashovers in medium-voltage metal-clad electrical equipment. The root cause of this issue is an electrical breakdown of the air due to electrical stress and poor air quality within the switchgear. Without proper preventative measures, a flashover can occur, resulting in serious harm to workers and equipment. As a result, detecting corona faults in switchgear and preventing electrical stress buildup in switches is critical. Recent years have seen the successful use of Deep Learning (DL) applications for corona and non-corona detection, owing to their autonomous feature learning capability. This paper systematically analyzes three deep learning techniques, namely 1D-CNN, LSTM, and 1D-CNN-LSTM hybrid models, to identify the most effective model for detecting corona faults. The hybrid 1D-CNN-LSTM model is deemed the best due to its high accuracy in both the time and frequency domains. This model analyzes the sound waves generated in switchgear to detect faults. The study examines model performance in both the time and frequency domains. In the time domain analysis (TDA), 1D-CNN achieved success rates of 98%, 98.4%, and 93.9%, while LSTM obtained success rates of 97.3%, 98.4%, and 92.4%. The most suitable model, the 1D-CNN-LSTM, achieved success rates of 99.3%, 98.4%, and 98.4% in differentiating corona and non-corona cases during training, validation, and testing. In the frequency domain analysis (FDA), 1D-CNN achieved success rates of 100%, 95.8%, and 95.8%, while LSTM obtained success rates of 100%, 100%, and 100%. The 1D-CNN-LSTM model achieved a 100%, 100%, and 100% success rate during training, validation, and testing. Hence, the developed algorithms achieved high performance in identifying corona faults in switchgear, particularly the 1D-CNN-LSTM model due to its accuracy in detecting corona faults in both the time and frequency domains.

## 1. Introduction

Switchgear plays a crucial role in power distribution networks [1]. The term “switching gear” refers to a variety of switching devices that perform functions such as controlling, protecting, and isolating power systems. This definition also encompasses devices used to regulate and meter power systems, as well as circuit breakers and other similar technologies [2]. Switchgear plays a critical role in ensuring the safety of downstream maintenance, including fault clearing, upkeep, equipment substitutions, and a range of other related concerns, especially in power-generating systems. It functions by isolating and de-energizing specific electrical components and networks to prevent potential hazards.

Furthermore, switchgear is typically classified into low, medium, and high voltage groups and is primarily constructed using insulating mediums such as air or oil [3]. It is crucial to closely monitor the performance and condition of switchgear during operation. Early detection of malfunctioning switchgear and prioritization of corrective maintenance are essential [4]. Failure to do so could result in negative outcomes for the distribution network, operational employees, and end users, leading to disruptive and unsettling fluctuations in customer data and regulatory perceptions [5]. 

Breakdowns in switchgear can be classified into several types, including arcing, tracking, surface discharge, and mechanical failure [6]. Corona discharge is a common issue that occurs in switchgear [7]. It is considered one of the primary causes of equipment breakdown, aging, and power loss in steady power systems [8]. In the event of an incident involving high-voltage systems, corona discharge faults can damage Medium-Voltage (MV) switchgear, which may result in equipment failure. MV switchgear is a crucial component of the power distribution network [9].

To maintain switchgear safety and ensure a stable power supply, it is crucial to detect and closely monitor corona discharge activities [10]. A corona discharge in switchgear results in the release of three types of energy: electromagnetic radiation, ultrasonic waves, and gases such as ozone and Nitrogen Oxides (NOx) [11]. Electromagnetic and ultrasonic techniques have been utilized to detect and monitor corona discharge activity in medium-voltage switchgear with reduced interference and improved efficiency [11]. In particular, electricity-based methods are commonly employed to measure discharge activities.

Offline detection procedures can be costly, and it can be challenging to identify the substances that promote corona discharge. To address this, research has investigated measuring ozone emissions post-discharge for fault identification and monitoring [12,13]. As a result, deep learning has gained significant traction and advancements in recent years and is now widely used across a wide range of industries, particularly in image and natural language analysis [14,15,16].

Convolutional neural networks (CNNs) are a popular type of deep learning model that can extract many features using various convolutional layers, including max pooling, normalization, and fully connected layers. [17]. However, CNNs are not suitable for remembering past time-series patterns. Recurrent neural networks (RNNs) are a specific type of neural network that can remember information from the past by using previous outputs as inputs [18,19].

Lately, several research studies have employed RNNs in the fields of speech recognition and natural language processing, among others. Specifically, the long short-term memory (LSTM) RNN architecture has been acknowledged and applied in time series processing. Its distinctive design explicitly resolves the problem of gradient vanishing in traditional RNNs and enables the learning of long-term dependencies, leading to more effective acquisition of the chronological aspects of sequential data [16,17,18,19,20,21]. 

The combination of CNN and RNN in a convolutional LSTM neural network is essential to model chronological sequences and improve deep neural network modeling capabilities. This network has been used in various large vocabulary tasks and has been shown to provide a relative improvement of 4–6% over an LSTM [22,23]. Additionally, there are many other studies focused on extracting temporal and spatial characteristics by combining CNN and LSTM models [24]. Table 1 presents the advantages and disadvantages of three methods that are commonly used in deep learning: 1D-CNN, LSTM, and 1D-CNN-LSTM. Each method has its own strengths and weaknesses, and researchers often choose one over the others depending on the specific task at hand. By comparing and contrasting these methods, researchers can determine which one is the best fit for their particular problem.

The following points summarize the study’s motivations and contributions:

1. This study uses deep learning techniques to detect corona faults in switchgear. The techniques employed include 1D-CNN, LSTM, and a hybrid model of 1D-CNN and LSTM;
2. The purpose of the research is to determine the most effective method for detecting corona faults. The hybrid method (1D-CNN-LSTM) was found to be the most effective compared to other methods;
3. The study is unique in that it was conducted in both the time and frequency domains, which had not previously been explored in this field;
4. The proposed models are able to quickly identify and differentiate corona faults from other types of faults. Overall, the hybrid model is considered the best model for detecting corona faults in both the time and frequency domains.

The categorization of the article can be seen; thus, Section 2 comprises the theoretical study and methods. Section 3 comprises performance metrics, Section 4 comprises results and discussion, and Section 5 concludes the paper.

## 2. Theoretical Study and Methods

### 2.1. The Theory of Corona Discharge

**Figure 1:**
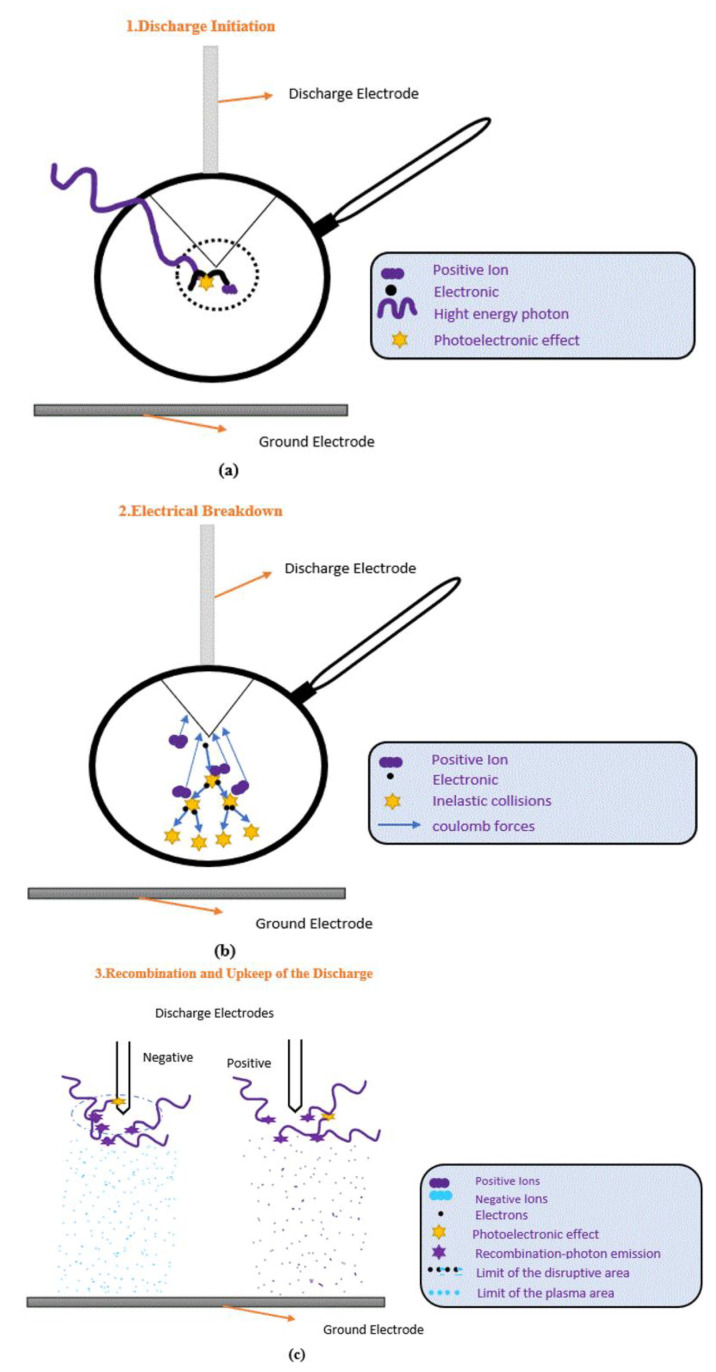
(**a**–**c**) A corona discharge is a phenomenon that occurs in the air [2].

A corona discharge refers to the phenomenon where a fluid, such as the air surrounding an electrically charged conductor, ionizes, and creates an electrical discharge. Without measures to reduce the electric field strength, high-voltage equipment may experience spontaneous corona discharges. A corona is formed when the electric field strength around a conductor is strong enough to create a conductive zone but not strong enough to cause electrical breakdown or arcing. Nevertheless, corona discharge can have detrimental effects on high-voltage equipment and cause a loss of power [2]. Figure 1a–c illustrates the corona discharge phenomenon and how the conductance and avalanche processes in Medium-Voltage (MV) switchgear are influenced by the electrode material. 

Figure 1 shows that when a neutral atom or molecule is exposed to a high electric potential gradient, it becomes ionized and initiates the corona discharge process. High-conductivity materials can lead to severe corona discharge flaws due to the strong electric field they produce, which attracts a lot of free electrons. Copper (Cu) and Stainless Steel (SS) are two popular metals used in MV switchgear due to their durability and conductivity. Copper has a higher conductivity than SS, with a value of 5.96107 Siemens per meter (S/m) compared to 1.45 × 106 (S/m) for SS. However, copper can produce higher corona discharges in MV switchgear than SS due to its higher conductivity.

Furthermore, when subjected to high-voltage stress, air, which contains electrically neutral molecules, acts as an insulator. When powerful energy travels as electrons from an electrode and strikes molecules, the molecules change [36].

The energy absorbed by the electrons surrounding atomic nuclei can lead to high-energy transformations, causing them to dissociate from the atoms or molecules they were attached to. This results in the formation of a positively charged ion and a dissociated free electron. Through the avalanche process, new electrons are produced as a result of electrons being struck by other electrons, leading to a mixture of electrons and ions [37], whereas the appearance of light signals from locally applied electric fields that permit sustained discharge into the atmosphere is another cause of corona discharge [38].

### 2.2. Proposed Methods

**Figure 2:**
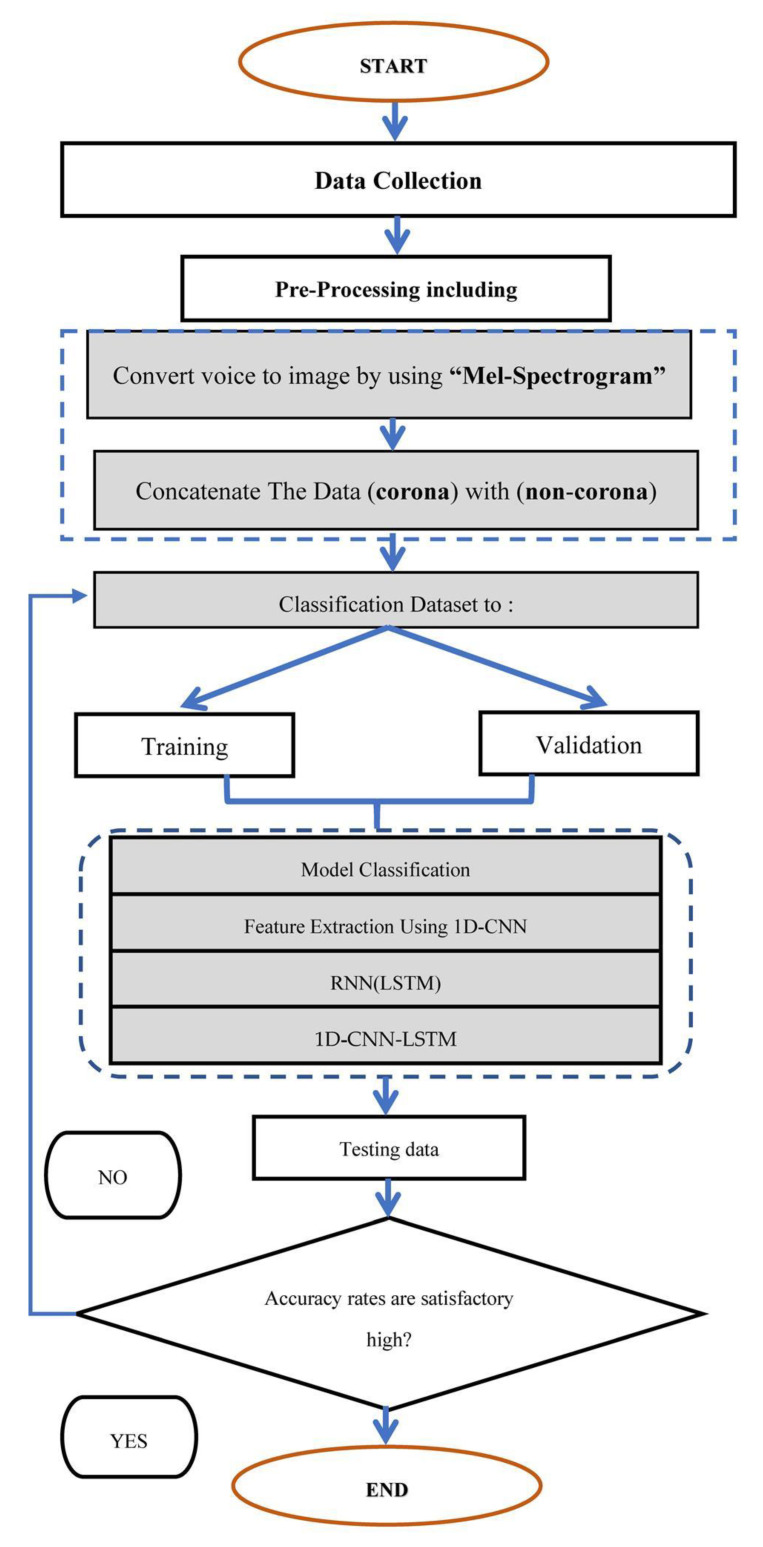
General flowchart diagram of the research method.

The proposed method for detecting corona faults involves collecting and preprocessing data in both the time and frequency domains. Figure 2 is used to illustrate the mechanism of data collection and preprocessing. The data is cleaned and collected, and then converted from sound to image using a mel-spectrogram. The data includes both corona and non-corona faults that occur in the switchgear, and it is classified into three phases: the training phase, the validation phase, and the testing phase. Features are then extracted using 1D-CNN, and classification is applied using the LSTM algorithm to achieve the highest possible accuracy. These methods are aimed at detecting corona faults in switchgear in both the time and frequency domains.

#### 2.2.1. Data Collection 

The test findings received using airborne ultrasonic testing (AUT) equipment were converted into raw data. The waveform audio file format, also known as wav, or the Moving Picture Experts Group (MPEG), was used to record the ultrasonic data. A technique for data preprocessing that consolidates or transforms the data into a format appropriate for a particular machine learning algorithm will be introduced as a result of this. The data, which were in the wav and mp3 file formats, were transformed into a matrix format to meet the requirements of the MATLAB software.

#### 2.2.2. Preprocessing 

The preprocessing step involves several stages, such as converting the raw audio data in wav or mp3 format to a matrix format suitable for the MATLAB programming language. The next step is to concatenate the corona with non-corona data, which includes other fault types like arcing, tracking mechanical, and normal, to differentiate between corona and other faults. Once the data is prepared, it is classified into three phases: training, validation, and testing, with 70%, 15%, and 15% of the data, respectively. Additionally, the “Mel-Spectrogram” technique is used to convert the voice data into an image format.

#### 2.2.3. D CNN 

**Figure 3:**
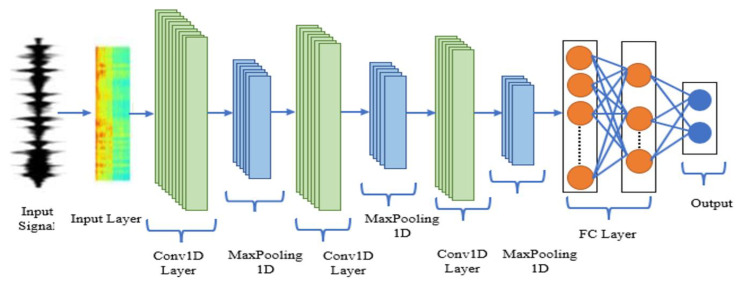
The basic structure of 1D-CNN.

A one-dimensional convolutional neural network (1D-CNN) is a neural network that operates on one-dimensional data, such as time series or sequence data. In this study, 1D CNNs are used to extract representative properties from corona and non-corona faults in both the time and frequency domains. This is achieved through 1D convolution operations using filters. Figure 3 shows the CNN structure, which includes convolutional layers, filters, a MaxPooling1D layer, a FC layer, and a classification layer with a Rectified Linear Unit (RELU) as the activation function. Dropout and batch size are used to avoid overfitting. In Equations (1–3), these equations are stated. (RELU), which can cause nonlinearity, is used as a CNN activation function. While deep CNNs were initially used for image classification tasks, they have since been used for video analysis and recognition. However, the use of 1D sequence data for classification and prediction is relatively new [2,3,4,5,6,7,8,9,10,11,12,13,14,15,16,17,18,19,20,21,22,23,24,25,26,27,28,29,30,31,32,33,34,35,36,37,38,39]. The corona and non-corona categories can be seen as a sequential modeling effort, making 1D CNNs a suitable choice. Compressed 1D-CNNs are preferred for real-time applications due to their low processing requirements [40].

(1) $$x_{o,fl}^{l}=f\left(\sum_{im}\;x_{i}^{l-1}*k_{io,fl}^{l}+b^{l}\right)$$

(2) $$x_{o}^{l}=f\left[max\left(\sum_{im}\;x_{i}^{l-1}\right)+b^{l}\right]$$

(3) $$x_{0}^{l}=f\left(x_{i}^{l-1}*d_{io}^{l}+b^{l}\right)$$

where x is a one-dimensional input matrix (n×1), f (.) is an activation function, $k_{io,fl}^{l}$ is a kernel filter (k×1),  lth is the number of filters F, the output of the lth convolutional layer is $x_{o,fl}^{l}$. b, denoted as bias vectors, and d are learnable parameters.

#### 2.2.4. RNN(LSTM) Structure

**Figure 4:**
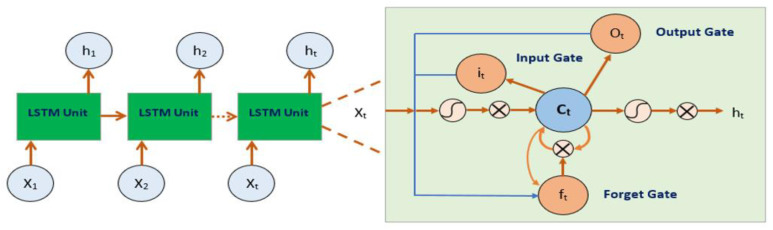
The basic structure of LSTM.

LSTMs (Long Short-Term Memory) are a type of RNN (Recurrent Neural Network) that overcome the vanishing gradient problem in traditional RNNs and are capable of capturing long-term dependencies in sequential data. In LSTMs, the hidden layers of RNNs are replaced with memory cells that are capable of selectively retaining or discarding information using gates. There are three types of gates in an LSTM: the input gate, the output gate, and the forget gate, which regulate the flow of information into and out of the memory cell [41]. For consecutive modeling applications like text categorization and time series modeling, LSTM is very useful [29] LSTM’s structural layout is drawn in Figure 4. The mathematical expression for an LSTM block presented by Equations (4)–(9) is as follows:

(4) $$i_{t}=\sigma \left(\;x_{t}U^{i}+h_{t-1}W^{i}+b_{i}\right)$$

(5) $$f_{t}=\sigma \left(\;x_{t}U^{f}+h_{t-1}W^{f}+b_{f}\right)$$

(6) $$O_{t}=\sigma \left(\;x_{t}U^{o}+h_{t-1}W^{o}+b_{o}\right)$$

(7) $$C_{t}=\sigma \left(\;f_{t}⊙C_{t-1}+i_{t}⊙\overset{ˇ}{C}{\;}_{t}U^{i}+b_{c}\right)$$

(8) $$\overset{ˇ}{C}=tanh\left(\;x_{t}U^{g}+h_{t-1}W^{g}\right)$$

(9) $$h_{t}=tanh\left(\;C_{t}\right)⊙O_{t}$$

where $x_{t}$ is the network input matrix, $h_{t}$ is the output of the hidden layer, and σ indicates the SoftMax function. C is a memory cell that facilitates recalling a significant case, which is counted using Equation (7). The elderly cell case $C_{t-1}$ is updated to the modern cell case $C_{t}$. New nominee values $\overset{ˇ}{C}$ and the output of the present LSTM block $h_{t}$ makes use of the hyperbolic tangent function as shown in Equations (8) and (9). The parameters for observation are weights ($W^{i}$, $W^{f}$, $W^{o}$, and $W^{g}$) and ($U^{i}$, $U^{f}$, $U^{o}$, and $U^{g}$) that create the neural network’s internal parameters and bias vectors ($b_{i}$, $b_{f}$, $b_{o}$, and $b_{c}$). The model improves weights and biases by reducing the goal function. The operator ⊙ denotes the multiplication of elements.

#### 2.2.5. D-CNN-LSTM

**Figure 5:**
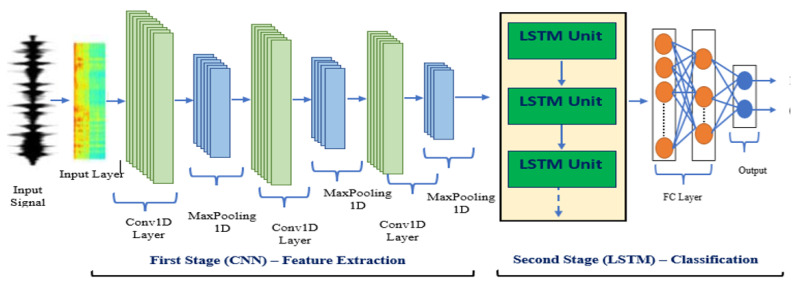
An instance of a 1D-CNN-LSTM model.

After the feature extraction stage by 1D-CNN, the output is fed into an LSTM layer, which can capture the long-term dependencies of the input sequence. The LSTM layer helps in learning the temporal relationship between the features extracted by the 1D-CNN model. The output of the LSTM layer is then passed through a fully connected layer for classification into corona or non-corona faults in both domains. The proposed hybrid model benefits from the strengths of both 1D-CNN and LSTM and is expected to achieve better performance in identifying corona faults compared to using either model alone as shown in Figure 5.

In the time domain analysis, the recording sampling comprises a multi-input, series-dimensional matrix. The second stage involves training and classifying corona and non-corona cases using the LSTM neural network. The Adam optimizer was used before the optimization process, which is a popular method for weight optimization. The model’s accuracy and losses have been studied by varying the number of convolutional layers, kernel size, MaxPooling1D, and Fully Connected (FC) layer as well as the model parameters, which have been optimized to improve efficiency. The samples of datasets for corona and non-corona in the time domain are in Table 2, which has a size of 17.5 megabytes (MB) in the time domain.

**Figure 6:**
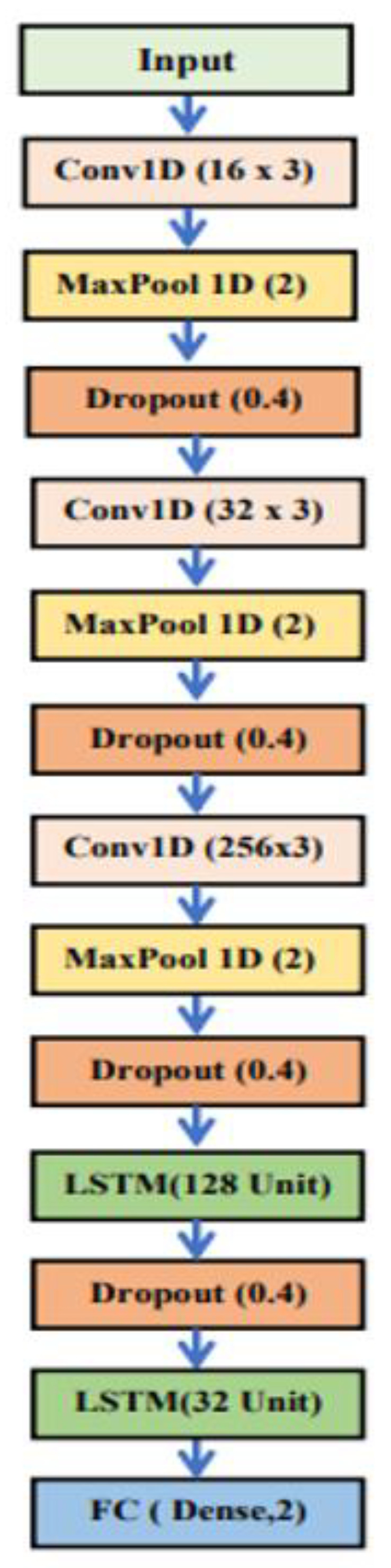
The suggested 1D CNN-LSTM model’s structure (time domain).

As a result, the 1D CNN-LSTM structure consists of three 1D convolutional layers and three global MaxPooling1D layers, with a 0.5% dropout for RELU to accelerate processing. MaxPooling1D is set to 2, and the kernel size is 3. Following these are two LSTM layers with 128 and 32 units, respectively, and a 0.5% dropout. The FC layer, with a SoftMax activation function, is shown below, and the learning rate is 0.0001 with a batch size of 16 and an epoch number of 100. Figure 6 depicts the CNN-LSTM architecture as designed.

On the one hand, the 1D CNN architecture previously described consists of two one-dimensional convolutional layers, each with 16 and 32 filters, respectively. A drop-out rate of 0.4 is applied after each layer, followed by two max pooling layers with a size and kernel established at 3 and 2. The fully connected layers use the SoftMax activation function with a learning rate of 0.0001 and batch size of 16, and the number of epochs is 60. The LSTM architecture for corona and non-corona fault detection consisted of two connected LSTM units with 32 and 32 LSTM units, resulting in a significant increase in training time. As a result, a fully connected layer of 32 neurons with a SoftMax activation function and a learning rate of 0.0001 was constructed, with a batch size of 16 and a number of epochs of 50. The detailed structural design of the deep learning algorithms can be found in Table 3. 

In the case of frequency domain analysis, the suggested method involves recording samples as dimensional matrices in the frequency domain. The samples are then used to teach an LSTM neural network how to tell the difference between cases of corona and non-corona, and the same steps are taken in the time domain where the Adam optimizer is used to optimize the weights of the neural network during training. The number of convolutional layers, kernel size, MaxPooling1D, and FC layer are all model parameters that are investigated to achieve the best possible accuracy and loss. By tweaking these parameters, the model’s performance can be improved. The samples of datasets for corona and non-corona in the frequency domain are shown in Table 4, which has a size of 11.3 megabytes (MB) in the frequency domain.

**Figure 7:**
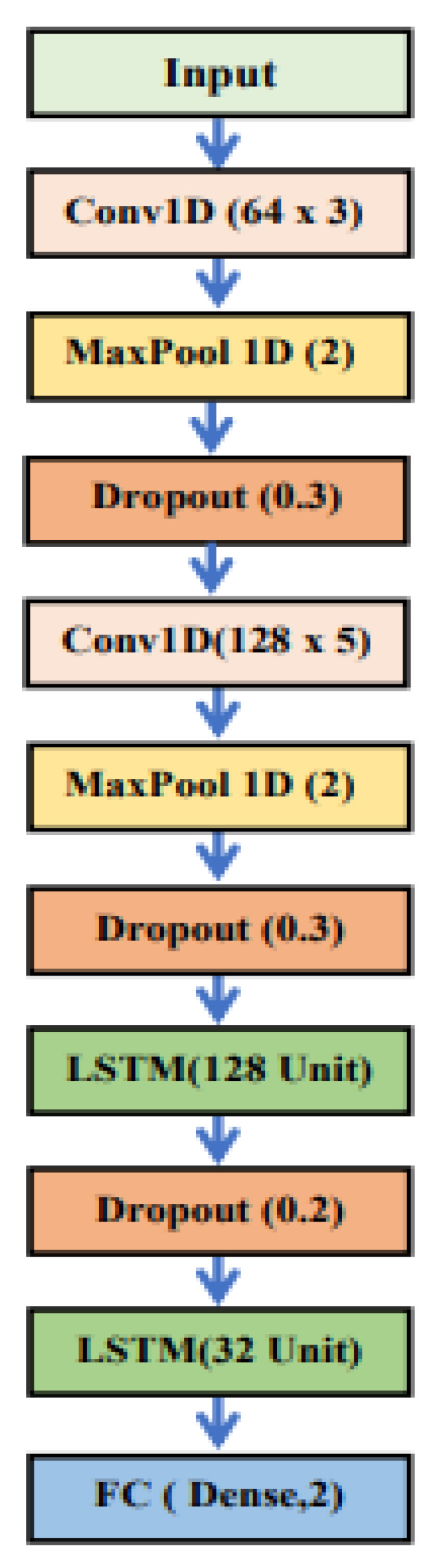
The suggested 1D CNN-LSTM model’s structure (frequency domain).

The 1D-CNN-LSTM model structure consists of two global max pooling layers and two 1D convolution layers. To save processing time, a dropout of 0.3 is employed after each layer from the global max pooling layers, with RELU activation used for the CNN layers. The MaxPooling1D is set to 2, and the kernel size is equal to 3. After the two CNN layers, there are two LSTM layers with 128 and 32 units, respectively, followed by a dropout of 0.3. The subsequent fully connected (FC) layer has a learning rate of 0.0001, a batch size of 16, and 100 epochs with a SoftMax activation function. The structure of the 1D-CNN-LSTM is depicted in Figure 7.

The 1D-CNN model architecture consists of three one-dimensional convolutional layers with 64, 128, and 256 filters for each layer. Three max pooling layers are also employed, with a dropout of 0.3 between each of them to prevent overfitting. The sizes of the kernel and MaxPooling1D are set to 3 and 2, respectively. Finally, a SoftMax activation function and learning rate of 0.0001 are used for the last layer of the network, as well as a batch size of 16 and a number of epochs of 60. Overall, this architecture appears to be a deep learning approach for classifying corona and non-corona cases based on time series data.

On the other side, the LSTM model consists of two connected LSTM units with 64 and 32 units, respectively, and a fully connected layer of 32 neurons. A fully connected layer with a SoftMax activation function, a learning rate of 0.0001, a batch size of 16, and a number of epochs of 50. The training time for this model was found to be significantly higher than the hybrid 1D-CNN-LSTM model. Table 5 provides detailed information on the designs of the deep learning algorithms used in this study. Overall, both the hybrid 1D-CNN model and the LSTM model and the hybrid 1D-CNN-LSTM model are deep learning approaches that are designed to classify corona and non-corona cases based on time series data. However, the specifics of the architectures differ, and their effectiveness and generalizability would depend on the specifics of the dataset and training process.

## 3. Performance Metrics

Because of this, it is difficult to assess the effectiveness of the suggested models in Table 6 using the paper confusion matrix. The situations of corona and non-corona are represented, respectively, by 1 and 0. Equations (8)–(11) provide a number of indices, including classification accuracy, dependability, sensitivity, and F1-Measure (11). The confusion matrix makes use of them to evaluate the model’s performance. The recall is the portion of a pertinent example that is remembered out of all the pertinent samples. Precision is the portion of the recovered samples that are pertinent samples. As a separate performance indicator, the cross-entropy loss is used to assess how accurately the model predicts the target data as provided in Equation (12). The reduction of the difference between the corona and non-corona condition probability distributions is a crucial performance parameter. Most issues involving binary classification employ it. For a decent model, the loss value is 0.

(10) $$Classification\;Accuracy\;\left(\%\right)=100\times \frac{TP+TN}{TP+FP+FN+TN}\;,$$

(11) $$Recall\;\left(Sensitivity\right)\left(\%\right)=100\times \frac{\mathrm{TP}}{\mathrm{TP}+\mathrm{FN}}\;,$$

(12) $$Precision\;\left(Dependability\right)\left(\%\right)=100\times \frac{TP}{TP+FP}\;,$$

(13) $$F1\;measure\;\left(\%\right)=100\times 2\times \frac{\left(Precision\times Recall\right)\;}{\left(Precision+Recall\right)\;}\;,$$

(14) $$Loss\;\left(L\right)=-\frac{1}{N}*\sum_{I=1}^{M}\;T_{I}\log\;\left(x_{i}\right).$$

In an instance in which $X_{i}$ is the predicting response, $T_{i}$ will be the target value, M will be the total number of responses predicting in X (all findings and classes combined), and N will be the cumulative number of observations in X. The binary cross entropy missed is totaled, inclusive of divisions and surveillance, and normalized by the number of states.

## 4. Results and Discussion

On the basis of the sound waves collected when the switchgear is in operation, experiments are carried out to illustrate how well the created algorithms function in locating corona faults in the switchgear. The tests are conducted in the time domain and the frequency domain, which are two distinct domains. For 1DCNN, LSTM, and 1D-CNN-LSTM, in order to distinguish between corona and non-corona faults in the time domain, a total of 438 data samples were created and split into training, validation, and testing datasets. The test dataset contained only positive or negative cases of corona. Seventy percent of the data was used for training, while fifteen percent was used for both validation and testing.

Each phase of the study consisted of 306 cases for testing, 66 cases for validation, and 66 cases for training. The confusion matrix presented in the tables below shows the true and false detection percentages based on the best results from repeated trials. Table 7 presents the results of the 1D-CNN-LSTM training phase in the time domain, using a total of 306 datasets. Among these, 26 cases of corona and 278 cases of non-corona were correctly identified. Only two corona cases were misclassified as non-corona, resulting in an accuracy of 99.3% and an error rate of 0.7%. On the other hand, the CNN accurately identified 274 non-corona cases and 26 corona cases, while misclassifying five corona cases as non-corona, resulting in an accuracy of 98.3% and an error rate of 1.7%. Similarly, the LSTM identified 26 corona cases and 272 non-corona cases, with eight corona cases misclassified as non-corona, resulting in an accuracy of 97.3% and an error rate of 2.7%.

During the validation stage of the time domain analysis for 1D-CNN-LSTM, 66 sets of data were used, including seven cases of corona and fifty-eight cases of non-corona. Unfortunately, one corona case was misclassified as a non-corona case, resulting in an overall accuracy of 98.4% with a 1.6% error rate. Both the CNN and LSTM models were employed during this analysis, and the results were similar. Seven corona cases and fifty-eight non-corona cases were successfully identified by both models, with one corona case being incorrectly classified as non-corona. The overall accuracy rate for both models was determined to be 98.4%, with a 1.6% error rate. Table 8 shows the detailed matrix results for this analysis.

The results of the testing are shown in Table 9. According to the information provided, the algorithm has used a dataset of 66 sets of data for testing and has achieved an accuracy of 98.4% with a 1.6% error rate in identifying non-corona faults and corona faults using 1D-CNN-LSTM. Specifically, out of the 66 sets of data, the algorithm has correctly identified fifty-seven non-corona faults and eight corona cases but has mistakenly recognized one corona case as non-corona. Furthermore, the algorithm has also been tested using only CNN and LSTM, separately. When using CNN alone, the algorithm has achieved an accuracy of 93.9% with a 6.1% error rate, and when using LSTM alone, the algorithm has achieved an identical accuracy and error rate. In both cases, the algorithm has identified fifty-three non-corona faults and eight cases of corona, but has misidentified five cases of corona as non-corona, while for CNN, the algorithm has identified fifty-four non-corona faults and eight cases of corona, but has misidentified four cases of corona as non-corona.

According to the information given in the frequency domain analysis, the dataset used in this analysis consisted of 160 samples, with 70% being used for training and 15% for both validation and testing. The training dataset comprised 112 samples, while the validation and testing datasets consisted of 24 and 24 samples, respectively.

The results of the validation phase are presented in confusion matrices for each method, showing the best and worst results from repeated experiments as well as the percentage of correct and false detections.

Table 10 shows the output matrix for the training phase of a deep learning algorithm using 1D-CNN-LSTM for corona and non-corona fault detection in the frequency domain. According to the information provided, the training dataset consisted of 112 sets of data, and the algorithm has identified 87 non-corona cases and 25 corona cases with 100% accuracy and a 0% error rate. The algorithm has also been tested using only CNN and LSTM, separately. When using CNN alone, the algorithm has identified 83 non-corona cases and 29 corona cases with 100% accuracy and a 0% error rate. When using LSTM alone, the algorithm has identified 83 non-corona cases and 29 corona cases with 100% accuracy and a 0% error rate.

The validation dataset in the frequency domain analysis consisted of 24 sets of data, and the 1D-CNN-LSTM algorithm has identified seventeen non-corona cases and seven corona cases with 100% accuracy and a 0% error rate. When using only CNN, the algorithm has identified nineteen non-corona cases and four corona cases with 95.8% accuracy and a 1.2% error rate, where one corona case was mistakenly classified as a non-corona case. When using only LSTM, the algorithm has identified sixteen non-corona cases and five corona cases with 100% accuracy and a 0% error rate. The resulting matrix is displayed in Table 11.

Based on the information provided, the 1D-CNN-LSTM algorithm achieved 100% accuracy and a 0% error rate in the testing phase of the frequency domain analysis with 24 sets of data, identifying seventeen non-corona cases and seven corona cases. When using only CNN, the algorithm identified nineteen non-corona cases and four corona cases with 95.8% accuracy and a 4.2% error rate, where one corona case was wrongly classified as a non-corona case. When using only LSTM, the algorithm identified nineteen non-corona cases and five corona cases with 100% accuracy and a 0% error rate, as shown in Table 12.

A thorough analysis using performance metrics, including sensitivity, dependability, loss, accuracy, and F1-Measure in Table 13 and Table 14, has been done to further study the truthfulness of CNN-LSTM.

Based on the results presented in Table 7, Table 8, Table 9, Table 10, Table 11 and Table 12, it can be concluded that both LSTM and 1D-CNN-LSTM models are effective in detecting corona and non-corona faults in both the time and frequency domains. In the testing phase, the 1D-CNN-LSTM model achieved higher accuracy (98.4%) compared to the LSTM model (92.4%) in the time domain analysis, while both models achieved 100% accuracy in the frequency domain analysis.

However, it is worth noting that in the time domain analysis, the 1D-CNN-LSTM model wrongly identified one corona case as a non-corona case, which led to a slightly lower accuracy compared to the frequency domain analysis. This suggests that further improvements can be made to the 1D-CNN-LSTM model to increase its accuracy in identifying corona cases.

Overall, the results indicate that the 1D-CNN-LSTM model is a promising approach for fault detection in power systems and has the potential to outperform traditional methods. The training process for the 1D-CNN-LSTM model in both the time and frequency domains is more time-consuming than the other techniques due to the large number of model parameters, as shown in Table 2 and Table 3. The 1D-CNN model is considered the most efficient and quickest for training time, with a total of 6738 parameters in the time domain. On the other hand, the LSTM model is considered the most efficient and quickest for training time in the frequency domain, with a total of 35,298 parameters.

It should be noted that the total time required for each model differs between the time domain and frequency domain analyses, as shown in Table 15 below. It is important to mention that the models were implemented on Google Colab, and the LSTM model required the least amount of time for implementation in the time domain analysis, while the 1D-CNN model required the least amount of time for implementation in the frequency domain analysis.

Upon analyzing the outcomes of the three phases of training, validation, and testing, we can conclude that the three models (1D-CNN, LSTM, and 1D-CNN-LSTM) exhibit superior performance compared to the results obtained by the study of [42]. Based on the results obtained from the three phases of training, validation, and testing, we can observe that the three models (1D-CNN, LSTM, and 1D-CNN-LSTM) outperform the results obtained by the researcher, who utilized the extreme learning machine approach to detect corona in the same data in both the time and frequency domains. In the time domain analysis, the results obtained were 90.63%, 87.5%, and 87.5%, while in the frequency domain analysis, the results were 89.84%, 83.33%, and 87.5%. Therefore, we can conclude that the hybrid model, which is the 1D-CNN-LSTM model, has proven its effectiveness in terms of accuracy and error rate in comparison to the previous techniques, and it is a suitable model for detecting corona in switchgear. 

## 5. Conclusions

In conclusion, switchgear plays a critical role in ensuring safety and protection, especially during downstream repairs. Inadequate monitoring, inspection, and evaluation can cause switchgear malfunctions, making it necessary to have a reliable flaw identification system to prevent manual inspection and aid in proper operation. This research proposes various essential deep learning methodologies for corona detection. CNN and LSTM algorithms have previously been used to classify datasets automatically, and 1D CNN, LSTM, and CNN-LSTM architectures were presented to determine defect detection systems based on sound waves in this study. Time and frequency domain analyses were conducted, and the success rates in differentiating corona and non-corona instances in the training, validation, and testing phases were evaluated. The 1D-CNN achieved success rates of 98.3%, 98.4%, and 93.9% in the time domain analysis, while the LSTM achieved 97.3%, 98.4%, and 92.4%. The best-performing method was 1D-CNN-LSTM, with success rates of 99.3%, 98.4%, and 98.4%. In the frequency domain analysis, the 1D-CNN achieved success rates of 100%, 95.8%, and 95.8%, while the LSTM achieved 100%, 100%, and 100%. The accuracy for 1D-CNN-LSTM and the success rates in the training, validation, and testing phases were 100%, 100%, and 100%, respectively. The proposed algorithm performed well and could be enhanced and implemented in real-time simulations, particularly in industrial sectors.

## Figures and Tables

**Table 1:** Summary of the advantages and disadvantages of the proposed methods.

Ref	Methods	Advantages	Disadvantages
[25] [26] [27] [28]	1D-CNN	Can learn complex patterns in data through feature extraction; Effective for processing sequential data; Can handle high-dimensional inputs; Can be computationally efficient.	Not ideal for handling variable-length inputs; May not perform as well as LSTM for tasks that require long-term memory.
[29] [30] [31] [32]	LSTM	Effective for processing sequential data and modeling long-term dependencies; Can handle variable-length inputs; Can retain and selectively forget information over long time intervals; Can be combined with other models, such as CNNs, for improved performance.	Can be computationally expensive; May be overfit on small datasets.
[33] [21] [34] [35]	1DCNN-LSTM	Can combine the strengths of both 1D-CNN and LSTM models for improved performance; Effective for processing sequential data and modeling long-term dependencies; Can handle high-dimensional and variable-length inputs.	Can be computationally expensive; May be overfit on small datasets; Overall, the choice of model will depend on the specific characteristics of the problem being solved and the available data.

**Table 2:** The datasets sample corona and non-corona in the time domain.

No	Name of Samples	No of Samples
1	Arcing faults	54 ∗ 20,001
2	Corona faults	41 ∗ 20,001
3	Tracking faults	313 ∗ 20,001
4	Mechanical faults	17 ∗ 20,001
5	Normal faults	13 ∗ 20,001

**Table 3:** The structural design for deep learning algorithms in the time domain.

1D-CNN	LSTM	1D-CNN-LSTM
1DConv (16) (RELU)	LSTM (32)	1DConv (16) (RELU)
Drop-out	Drop-out	MaxPooling1D
MaxPooling1D	LSTM (32)	Drop-out
1DConv (32) (RELU)	Drop-out	1DConv (32) (RELU)
Drop-out	Flatten	MaxPooling1D
MaxPooling1D	Dense (32) (RELU)	Drop-out
Flatten	Dense (2) (SoftMax)	1DConv (256) (RELU)
Dense (32) (RELU)		MaxPooling1D
Dense (2) (SoftMax)		Drop-out
		LSTM (128)
		Drop-out
		LSTM (32)
		Dense (2) (SoftMax)
The number of total parameters		
6738	16,226	245,170

**Table 4:** The samples of datasets for corona and non-corona in the frequency domain.

No	Name of Samples	No of Samples
1	Arcing faults	53 ∗ 10,001
2	Corona faults	39 ∗ 10,001
3	Tracking faults	40 ∗ 10,001
4	Mechanical faults	16 ∗ 10,001
5	Normal faults	12 ∗ 10,001

**Table 5:** The structural design for deep learning algorithms in the frequency domain.

1D CNN	LSTM	1D CNN-LSTM
1DConv (32) (RELU)	LSTM (64)	1DConv (64) (RELU)
Drop-out	Drop-out	MaxPooling1D
MaxPooling1D	LSTM (32)	Drop-out
1DConv (64) (RELU)	Drop-out	1DConv (128) (RELU)
Drop-out	Flatten	MaxPooling1D
MaxPooling1D	Dense (32) (RELU)	Drop-out
1DConv (1024) (RELU)	Dense (2) (SoftMax)	LSTM (128)
Drop-out		Drop-out
MaxPooling1D		LSTM (32)
Flatten		Dense (2) (SoftMax)
Dense (32) (RELU)		
Dense (2) (SoftMax)		
The number of total parameters		
143,650	35,298	197,250

**Table 6:** Confusion matrix for corona and non-corona cases.

	Predictive Corona Findings (1)	Predictive Non-Corona Findings (0)
Actual corona findings (1)	TP	FP
Actual non-corona findings (0)	FN	TN

The right forecast is shown as True Positive (TP) and True Negative (TN), while the wrong forecast is expressed as False Positive (FP) and False Negative (FN).

**Table 7:** Time domain corona fault classification output matrix for the training phase.

	1D-CNN		LSTM		1D-CNN-LSTM	
	Corona	Non-Corona	Corona	Non-Corona	Corona	Non-Corona
Actual corona	26	5	26	8	26	2
Actual non-corona	0	275	0	272	0	278

**Table 8:** Time domain corona fault classification output matrix for the validation phase.

	1D-CNN		LSTM		1D-CNN-LSTM	
	Corona	Non-Corona	Corona	Non-Corona	Corona	Non-Corona
Actual corona	7	1	7	1	7	1
Actual non-corona	0	58	0	58	0	58

**Table 9:** Time domain corona fault classification output matrix for the testing phase.

	1D-CNN		LSTM		1D-CNN-LSTM	
	Corona	Non-Corona	Corona	Non-Corona	Corona	Non-Corona
Actual corona	8	4	8	5	8	1
Actual non-corona	0	54	0	53	0	57

**Table 10:** Frequency domain corona fault classification output matrix for the training phase.

	1D-CNN		LSTM		1D-CNN-LSTM	
	Corona	Non-Corona	Corona	Non-Corona	Corona	Non-Corona
Actual corona	29	0	29	0	25	0
Actual non-corona	0	83	0	83	0	87

**Table 11:** Frequency domain corona fault classification output matrix for the validation phase.

Actual Class	1D-CNN		LSTM		1D-CNN-LSTM	
	Corona	Non-Corona	Corona	Non-Corona	Corona	Non-Corona
Actual corona	4	1	5	0	7	0
Actual non-corona	0	19	0	19	0	17

**Table 12:** Frequency domain corona fault classification output matrix for testing the phase.

	1D-CNN		LSTM		1D-CNN-LSTM	
	Corona	Non-Corona	Corona	Non-Corona	Corona	Non-Corona
Actual corona	4	1	5	0	7	0
Actual non-corona	0	19	0	19	0	17

**Table 13:** The assessment of various metrics in accordance with the DL structure’s testing-phase performance results for the time domain.

Case Corona Outcome (1)				
Approaches	Accuracy	Sensitivity	Dependability	F1-Measure
1D-CNN	92.4	100	62	76
LSTM	92.4	100	62	76
1D-CNN-LSTM	98.4	98	89	94
**Case Non-Corona Outcome (0)**				
**Approaches**	**Accuracy**	**Sensitivity**	**Dependability**	**F1-Measure**
1D-CNN	92.4	91	100	95
LSTM	92.4	91	100	95
1D-CNN-LSTM	98.4	98	100	99

**Table 14:** The assessment of different metrics in light of the best performance outcomes of DL structures during testing for the frequency domain.

Case Corona Outcome (1)				
Approaches	Accuracy	Sensitivity	Dependability	F1-Measure
1D-CNN	95.8	100	95	97
LSTM	100	100	100	100
1D-CNN-LSTM	100	100	100	100
**Case Non-Corona Outcome (0)**				
**Approaches**	**Accuracy**	**Sensitivity**	**Dependability**	**F1-Measure**
1D-CNN	95.8	100	80	89
LSTM	100	100	100	100
1D-CNN-LSTM	100	100	100	100

**Table 15:** The calculation times of the methods in the time and frequency domains.

Methods	Time Domain	Frequency Domain
1D-CNN	0:00:42 s	0:00:18 s
LSTM	0:00:26 s	0:00:21 s
1D-CNN-LSTM	0:00:39 s	0:00:28 s

## Data Availability

Not applicable.
